# Comparison of ^15^oxygen positron emission tomography and near-infrared spectroscopy for measurement of cerebral physiology

**DOI:** 10.1186/cc14525

**Published:** 2015-03-16

**Authors:** J Simpson, N Sudhan, H Hare, J Donnelly, X Liu, F Aigbirhio, T Fryer, G Stocks-Gee, P Smielewski, D Bulte, J Coles

**Affiliations:** 1 University of Cambridge, UK; 2 University of Oxford, UK

## Introduction

The gold standard technique for imaging cerebral blood flow (CBF) and metabolism is ^15^oxygen positron emission tomography (^15^O PET). Continuous near-infrared spectroscopy (NIRS) has been used to assess adequacy of cerebral oxygenation following stroke, traumatic brain injury and subarachnoid haemorrhage [1], and measurements have been compared with jugular oxygen saturation. In this study we compared NIRS with ^15^O PET within healthy volunteers.

## Methods

Fifteen healthy subjects were recruited (12 male, average age 38 years); PET precluded females of reproductive age. Steady-state ^15^O PET with arterial sampling was performed to measure CBF, cerebral metabolic rate of oxygen (CMRO_2_), oxygen extraction ratio (OEF) and cerebral blood volume (CBV) [2]. Simultaneously, NIRS data were collected using a Hamamatsu NIRO 200 with sensors on either side of the forehead. NIRS OEF was calculated from tissue oxygen saturation, SaO_2_ and an assumed arterial/venous blood volume ratio of 30/70 [3].

## Results

**Figure 1:**
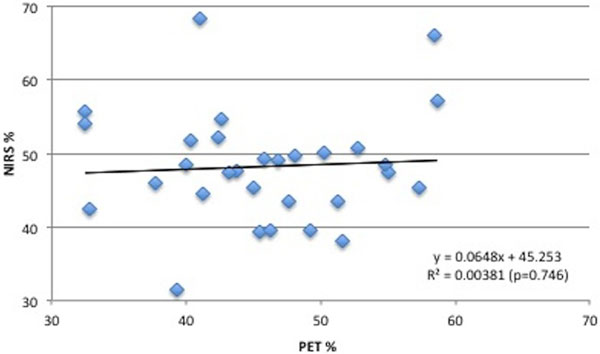
**Linear correlation between NIRS and PET OEF**.

The frontal region ^15^O PET CBF, CMRO_2_, OEF and CBV were mean (SD) 44.9 (10) ml/100 ml/minute, 158.7 (24.7) μmol/100 ml/minute, 45.8 (7.3)%, and 2.8 (0.8) ml respectively, and there was no relationship between NIRS and ^15^O PET (Figure 1).

## Conclusion

We found no relationship between NIRS and baseline physiology as determined by ^15^O PET. Further studies should assess the dynamic response of NIRS to a measured change in physiology in patients. Further confines of NIRS include its limited and focal brain coverage.
